# Spatio-temporal characterization of malaria prevalence in a peri-urban resource limited setting of Western Kenya Highlands

**DOI:** 10.4314/ahs.v24i3.14

**Published:** 2024-09

**Authors:** Beatrice Aleyo Muzame, Elizabeth Omukunda, David Mulama, Patrick Okoth

**Affiliations:** Department of Biological Sciences, School of Natural Sciences, Masinde Muliro University of Science and Technology (MMUST), P.O.Box 190-50100, Kakamega, Kenya

**Keywords:** *Plasmodium* species, space, time trend, malaria prevalence, socio-economic factors, demographic factors

## Abstract

**Background:**

Malaria is the main killer disease in sub-Sahara Africa.

**Objective:**

The study identified malaria prevalence patterns in relation to space and time trends in a peri-urban resource limited setting of Western Kenya highlands aimed at strengthening implementation of malaria control strategies.

**Method:**

A longitudinal study was carried out in Mbale town and its environs, Vihiga County, from December 2019 to November 2020. Among patients who presented themselves at Mbale Provincial Rural Training health center for different medical issues, 768 malaria confirmed patients were recruited and signed consent before the study commenced. Data was collected using questionnaire and microscopy which was presented through graphs, frequency, means and analyzed using linear regression. P-value ≤ 0.05 was considered statistically significant.

**Results:**

*Plasmodium falciparum* malaria constituted 98.7%,while *P. malariae*, *P. vivax*, and *P. ovale* constituted 1.3%. Linear regression analysis showed effect of age, gender and location on malaria prevalence as (R2 = 0.7, [F (3,764) = 1.854], p < 0 136). Malaria prevalence varied in different months due to changes in amount of rainfall and temperatures received.

**Conclusion:**

Spatio-temporal characterization and many mosquito breeding grounds influenced malaria prevalence in the study area. Malaria control strategies should be strengthened in relation to space and time-trend.

## Background

Spatio-temporal characterization of malaria prevalence in this study referred to how different geographical regions (space) and different time trends influenced malaria prevalence in the study area. Malaria is still a life-threatening and remains an acute public health problem especially in sub-Saharan Africa, despite various global intervention strategies having been put in place to reduce and finally eliminate it^1^. African Union has quality of life and well-being, health and economic growth as part of their 2063 agenda^2^, while health is one of Kenya's big four vision 2030 agenda^3^. In 2019, world health organization (WHO) reported that malaria prevalence and mortality had reduced significantly mainly in developing countries notably in Africa and aims at reducing malaria burden globally to 10% by the year 2030^4^. Female *Anopheles*' mosquitoes (*Anopheles gambiae, A. arabiensis* or *A. funestus*) take in human blood meal as they inoculate a malaria parasite that kills a child every minute globally^1^,^5^. *Plasmodium falciparum, Plasmodium vivax, Plasmodium ovale*, and *Plasmodium malariae*, which cause malaria infection in humans vary in space, time trend, severity, microscopic appearance, ability to cause relapses (*P. vivax* and *P. ovale*) and ability to develop resistance to antimalarial drugs^1^,^6^. About 99% of malaria globally is *Plasmodium falciparum* malaria^1^,^7^,^8^. The study's objective was to identify malaria prevalence patterns in relation to specific geographical locations (space) and time trends in the study area, aimed at strengthening implementation of malaria control strategies. The study area has been characterized with high malaria prevalence and mortality despite various malaria control strategies having been put in place^9^,^10^,^11^.

Malaria targeted risk populations that have low body immunity. These include; pregnant women, infants, children below the age of five years, the elderly, HIV and AIDS patients and travelers to malaria endemic regions^1^. Malaria prevalence varies in relation to; geographical location, time trend, age, gender and socio-economic factors according to study findings carried out in various parts of Kenya^1^, ^3^, ^7^ and other parts of the world^1^,^8^. Socio-economic factors tend to determine malaria transmission in a given area^1^. Socio-economic factors vary in different geographical regions which may influence economic activities and in turn influence malaria prevalence^1^, ^3^. Overstretching of resources due to high population may influence socio-economic status of people in a given area, which may affect malaria management^2^, ^3^. Socio-economic factors that are linked to malaria prevalence include; level of formal education, house designs, salary, household size, land use, wealth, sociocultural norms and expectations from women which exposes them to mosquito bites^1^, ^2^, ^9^. Poor perceptions and knowledge towards malaria management are to some extent associated with low level of formal education and poverty which is experienced in the highly populated regions in Kenya including the study area where dependency ratio is 100.90 with poverty level of 62%^3^.

Time trend and space influence economic activities and environmental factors which determine *Anopheles* mosquito breeding grounds and in turn malaria prevalence^12^, ^13^. High humidity accelerates the growth of malaria parasites in the *Anopheles* mosquitoes and enables mosquitoes to live longer, increasing malaria prevalence^1^, ^9^,^12^. Increase of temperature by 10°C tends to increase mosquito abundance and shorten incubation period of malaria parasites which increase malaria prevalence by 27%^1^. Effect of rainfall on malaria transmission is complex because both too little and too much rainfall tends to reduce mosquito population because too much rainfall can flush away mosquito breeding grounds while too little rainfall reduces breeding grounds for mosquitoes leading to decrease in malaria prevalence^9^. Effect of rainfall on malaria prevalence is observed 1-2 months after the rainfall because time taken from infective bite by *Anopheles* mosquitoes and appearance of the first symptoms of malaria is 7 to 30 days depending on the *Plasmodium* species^1^, ^4^.

Studies on effect of space and time trend on malaria prevalence were carried out in various parts of the world which included; Colombia Pacific Coast^4^, Malawi^14^, Mvomera district in Tanzania^15^, two high-risk districts of Nepal^16^, Tak Province in Thailand^17^, Amhara Region State in Ethiopia^18^, Gwanda district, Matabeleland in South Zimbabwe^19^, Sudan^20^ and Diebougou health district in Burkina Faso^10^ to assess whether malaria prevalence was increasing, decreasing or remained the same in different geographical regions, at different times to provide cost-effective malaria intervention strategies. A research carried out in Malawi^14^, urban Dares Salaam in Tanzania^21^ and a low endemic area of Eastern Rwanda^22^ showed decrease in malaria prevalence because the study was able to identify geographical distribution and time trends of malaria cases and implemented effective malaria control strategies. Studies on how space and time trend influence malaria prevalence were also carried out at Kenyan Coast^23^ and in lowlands and highlands of Western Kenya which included the following Counties; Homa Bay, Siaya, Vihiga, Kisumu, Kericho, Nandi and Bomet^11^, ^12^, ^24^, ^25^. Results from these studies indicated that malaria prevalence was still high despite intensive malaria control strategies having been put in place^26^. A study carried out on effect of climate on the spread of malaria in Vihiga County showed time trend had an impact on malaria prevalence^13^. This study will enable malaria case management bodies to predict malaria patterns in relation to specific geographical regions and time trends and plan for strengthening implementation of cost-effective malaria intervention strategies.

## Materials and Methods

### Study Site

The study area was Mbale town and its environs which covered three geographical locations which are currently referred to as wards in Kenya (Central Maragoli ward, Edzava ward and Lugaga/Wamuluma ward). The mapping of the study area was identified by a global positioning system (GPS) at Vihiga County headquarters. A baseline survey was done to familiarize with various mosquito breeding grounds in the study area before the study commenced. Mbale town is the headquarters of Vihiga County, found within Vihiga and Sabatia sub-Counties, Western Kenya, along Kisumu-Kakamega Road. It is located at latitude 0° 0′54.0′N and longitude 34°43′17.0 E with an altitude of 1200 metres above sea level, rainfall of between 1800mm to 2000mm annually, highest in April (288 mm) and lowest in January 94 mm,^27^,^28^. Highest average temperatures occur in February (21.4°C) and lowest in July (19.1°C) with an annual average temperature of 20.4°C^13^. The population in the study area was 60,000 people according to 2019 census and Maragoli as the predominant tribe^29^.

The main economic activities in the study area included; crop farming, livestock keeping, fishing, brick-making, mining and trade^27^. This study area was chosen because it was previously characterized for high malaria prevalence despite various malaria control strategies having been put in place^13^. Out of the patients who presented themselves at Mbale Provincial Rural Training Health Center for different medical issues, the study recruited 768 microscopically confirmed malaria patients who volunteered to participate in the study instead of 38230, 31, 32 33, to cover a large number of malaria confirmed patients in a densely populated malaria endemic study area.

### Inclusive and Exclusive Criteria

The study was purposive and equal numbers of 192 malaria confirmed patients or guardians who volunteered to participate in the study were recruited for each of the following age groups; children below 5 years old, children between 5-14 years old, children between 14-18 years old and adults. Malaria patients with; allergy to antimalarial drugs, chronic infections and people who had been on antimalarial treatment within the preceding 2 weeks were excluded from the study.

### Sample Size Determination

The sample size formula below was adopted from Krejcie and Morgan, 1970 32

S = X2NP(1-P)/d2(N-1) +X2P(1-P)
S =Desired sample size
X = Z-value = 1.96 for 95% confidence level, N = Population size (60,000)
P = Population proportion = 0.5.
d = degree of accuracy = 0.05.
S = (1.96)2(60,000) (0.5) (0.5)/(0.05)2(59,999) + (1.96)2(0.5) (0.5)
S = 382.

### Study Design and Study Period

The study adopted a longitudinal study design^30^, which was carried out from December 2019 to November 2020 to cater for both short and long rainy seasons.

### Data Collection

At the triage room of Mbale Provincial Rural Training Health Center, anthropometric characteristics of the study population were recorded by health workers^33^. Patients who were observed with signs of malaria by doctors at the health facility used were screened for malaria by qualified laboratory technicians using microscopy and observation according to world health organization guidelines^8^. Microscopy diagnosis by qualified laboratory technicians at the chosen health center was used to confirm malaria positive patients and identify *Plasmodium* species according to world health organization guidelines^8^. The area for finger-prick to collect blood was sterilized with methylated spirit and allowed to dry before blood collection was done. A blood film was air dried, fixed with methanol (thick film was not fixed), dried for 1 minute using a fan heater, stained with 10% giemsa stain for 10 minutes, giemsa stain cleaned with tap water, put in a water buffer of pH 7.2, immersed in oil and observed under 100 × microscope objectives to examine different *Plasmodium* species and quantify malaria parasites. Quantification of malaria parasites was carried out using WHO guidelines (8), which ranged from 120p/ul to 200000p/ul in the study population. Records on malaria prevalence data was obtained from the Mbale Provincial Rural Training Health Center.

#### Thick film


$$\frac{\text{Malaria}\;\text{parasites}\;\text{counted}}{200\;\text{white}\;\text{blood}\;\text{cells}}\times 8000=\text{parasites}/\text{ul}$$

#### Thin film


$$\frac{\text{Parasitized}\;\text{red}\;\text{blood}\;\text{cells}}{4000\;\text{red}\;\text{blood}\;\text{cells}}\times 5,000\;000=\text{number}\;\text{of}\;\text{parasites}/\text{ul}$$

The malaria patients and guardians who volunteered to participate in the study had their confidentiality guaranteed by using numbers instead of their names and signed the consent forms before the study commenced. Health workers (33) from the chosen health facility used interviews to administer semi-structured questionnaires to malaria patients, stratifying them into age, gender, geographical settings (wards) and socio- economic status according to Centers for Disease Control and Prevention guidelines (1).

### Ethical considerations

Masinde Muliro University of Science and Technology Institutional Ethical Review Committee granted an ethical review letter (MMU/COR: 509099) while National Commission for Science, Technology and Innovation provided permit (License number: NACOSTI/P/20/3379) for data collection. A written informed consent was signed by adult malaria patients and parents or guardians signed for malaria patients below 18 years old before the study commenced and their confidentiality was guaranteed.

### Data management and analysis

Data collected from the health facility used was entered in excel spreadsheets where it was checked, cleaned of all errors and analyzed using a SPSS software version 17. Means of malaria parasite species in the study population was compared using frequency tables. Linear regression was used to determine effect of geographical location (wards), gender, age and socio-economic factors on malaria prevalence while line graphs were also used to determine effect of time trend on malaria prevalence. Bar charts and line were used to determine associations between various socio-economic factors with malaria prevalence. P-value ≤ 0.05 was considered statistically significant in all tests.

## Limitations of the Study

The research was conducted during the COVID-19 pandemic period. Most patients were reluctant to visit health facilities during this period for fear of being infected with COVID-19. However, the data was collected although it took a long time because there were days when very few patients visited the chosen health facility during COVID-19 pandemic period.

## Results

From the microscopic malaria diagnosis results obtained, the distribution of malaria prevalence varied in different geographical locations (Wards) in the study area, which could have been due to difference in the availability of mosquito breeding grounds. Distribution of *Plasmodium* species in the study area was as follows; out of 768 malaria patients who were recruited in the study, *P. falciparum* malaria constituted 98.7% while *P. malariae* 0.8%, *P. vivax* 0.4% and *P. ovale* constituted 0.1%. From the results obtained, Lugaga/Wamuluma ward recorded 45.05%, malaria patients, Edzava ward 36.19% and Central Maragoli ward 18.75% (Table 1). All the three wards recorded *P. falciparum* malaria as the most common type of malaria infection in the study area. Edzava ward recorded all the four *Plasmodium* species while Central Maragoli and Lugaga/Wmuluma did not record *P. ovale* and *P. vivax* malaria (Table 1).

**Table 1:** Distribution of *Plasmodium* Species among Different Age Groups, Gender and Wards in the Study Area

	Plasmodium species				Total

*P. falciparum*		*P. malariae*	*P. ovale*	*P. vivax*	
Geographical Locations					
(WARDS)					
Central Maragoli	143	1	0	0	144
Edzava	272	2	1	3	278
Lugaga/Wamuluma	343	3	0	0	346
Total	758	6	1	3	768
AGE					
0-5 years old	188	3	0	1	192
5-14 years old	189	2	1	0	192
14-18 years old	191	0	0	1	192
Adults	190	1	0	1	192
Total	758	6	1	3	768
GENDER					
Male	366	2	1	2	371
Female	392	4	0	1	397
Total	758	6	1	3	768

*Plasmodium* species occurrence and *P. falciparum* prevalence varied by age (Table 1). Children below 5 years old and adults recorded *P. falciparum, P. malariae* and *P. vivax* malaria. Children aged 5-14 years old recorded *P. falciparum, P. malariae*, and *P. ovale* malaria. Children aged 5-14 years old were the only ones who recorded *P. ovale* malaria but not *P. vivax* malaria. Children aged 14-18 years old recorded *P. vivax* and recorded the highest *P. falciparum* malaria in the study area (Table 1). The study determined, if gender had any effect on distribution of *Plasmodium* species in the study area. From the results obtained, 49.3% malaria patients were male, while 51.7% were female (Table 1). *P. ovale* malaria was not recorded among female malaria patients, while all *Plasmodium* species were recorded among male malaria patients (Table 1).

From the linear regression analysis obtained, independent variables (wards, age and gender) showed the following effect on malaria prevalence (R^2^ = 0.7, [F (3,764) = 1.854], p < 0.136). Wards (space) had a statistically significant effect on malaria infection (P = 0.034) as opposed to gender (P = 0.321) and age (P = 0.712) as shown in Table 2. Distribution of malaria prevalence in the study area varied in relation to; space (Geographical location), age and gender as shown in Table 2.

**Table 2:** Linear Regression Showing effect of Wards, Age and Gender on Malaria Prevalence in the Study area Coefficients^a^

Model	Unstandardized Coefficients		Standardized Coefficients	T	Significance

	B	Std. Error	Beta		
(Constant)	351.207	39.242		8.950	.000
Ward	22.452	10.600	.076	2.118	.034
Gender	-15.893	16.006	-.036	-.993	.321
Age	2.637	7.153	.013	.369	.712

a. Dependent Variable: Malaria Prevalence

b. Predictors: (constant), Ward, Gender, Age

From linear regression analysis obtained, socio-economic factors had a statistically significant effect on malaria prevalence (R2 = 0.061, [F (7,760) = 7.063], p < 0.000). Individual socio-economic factors that were statistically significant to malaria prevalence in the study area were; level of education (p-value = 0.002), wealth (p-value = 0.000, size of land (p-value = 0.006, house type (p-value = 0.000 and ventilation in the house (p-value = 0.048) as opposed to salary (p-value = 0.828) and household size (p = 0.916) as shown in Table 3. Socio-economic factors tend to determine malaria transmission and they are likely to have influenced malaria prevalence in the study area.

**Table 3:** Linear Regression Results Showing Effect of Socio-economic Factors on Malaria Prevalence in the Study Area

Coefficients^a^						
Model		Unstandardized Coefficients		Standardized Coefficients	T	Sig.

		B	Std. Error	Beta		
	(Constant)	518.597	68.263		7.597	.000
	Level of education	22.966	7.562	.107	3.037	.002
	Salary	2.776	12.785	.009	.217	.828
	Wealth	-53.833	12.264	-.176	-4.389	.000
1	Size of land	15.507	5.592	.099	2.773	.006
	Household size	-.967	9.156	-.004	-.106	.916
	House type	-37.786	10.441	-.152	-3.619	.000
	Ventilation in the House	-37.454	18.883	-.083	-1.984	.048

a. Dependent Variable: + Malaria Prevalence

b. Predictors: (Constant), Ventilation in house, Household size, Level of education, Salary, Size of land, Wealth, House type.

Most individuals in the study area had moderate number of assests which accounted for 47.4 % with Lugaga/Wamuluma recording more moderate number of assests, followed by Edzava and lastly Central Maragoli (Table 4). Individuals who recorded large number of assests accounted for 25.9 % in the study area, who were more in Lugaga/Wamuluma ward and less in Central Maragoli ward, while individuals who recorded very few assests which accounted for 26.7 % in the study area, were less in Edzava ward as compared to the other two wards (Table 4). Sources of stagnant water which provided breeding grounds for mosquitoes in the study area included; poor disposal of empty containers, fish ponds, cracks in rocks, pits (tyre tracks) and old vehicle tyres found at car washing, burrow pits formed by brick- making and gold mining, swamps along streams, ditches formed by construction of buildings and roads. 46.2% of the study population named only one mosquito breeding ground correctly, which might have increased malaria prevalence in the study area.

**Table 4:** The Head of Household's Wealth Distribution and Information on Mosquito breeding Grounds in Different Geographical Locations (Wards) in the Study Area

	Geographical Locations (Ward)			
	Central Maragoli	Edzava	Lugaga/Wamuluma	Total
**Wealth (Assets)**				
Very few	41	81	83	205
Moderate	70	121	173	364
Large	33	76	90	199
**Total**	**144**	**278**	**346**	**768**
**Correctly named**				
**Mosquito breeding**				
**Grounds**				
One	63	127	165	355
Two	79	146	172	397
Three	2	3	9	14
Four	0	2	0	2
**Total**	**144**	**278**	**346**	**768**

**Figure 1:**
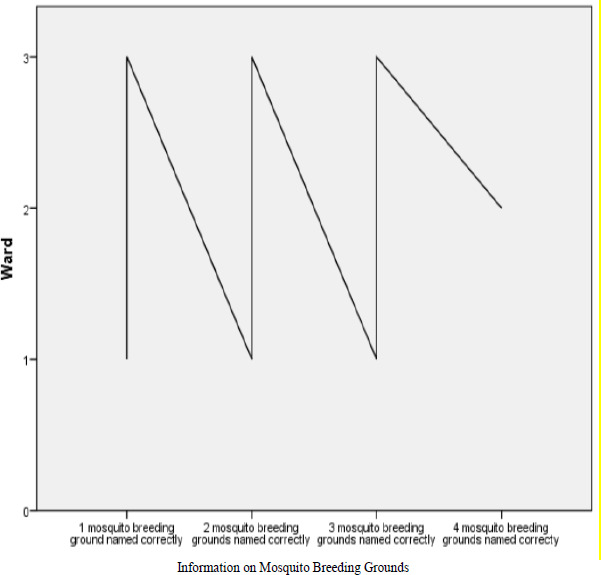
The Head of the Homestead's Information on Mosquitoes' Breeding Grounds in the Study Area (Wards: 1. Central Maragoli, 2. Edzava, 3. Lugaga/Wamuluma)

Records obtained from the study area indicated that, out of these 46.2% study population that was unable to correctly name more than 1 mosquito breeding grounds, Central Maragoli accounted for 17.75%, Edzava accounted for 35.77% while Lugaga/Wamuluma accounted for 46.48% (Figure 1).

Distribution of salary among study population in the wards found in the study area was high among individuals earning between Ksh.10, 000 to Ksh. 20,000 per month (51.4 %) who were considered to be earning moderate salary, followed by those who were considered to have had low salary of below Ksh. 10,000 per month (27.9 %), while those who earned high salary of over Ksh. 20,000 per month accounted for (20.7 %) as shown in Table 5. Lugaga/Wamuluma ward recorded highest number of individuals who earned high, moderate and low salary while Central Maragoli recorded the lowest (Table 5). Study population who recorded two household size accounted for 4.7 % with Edzava ward leading while Central Maragoli recorded the lowest, three household size accounted for 11.2 %, four household size accounted for 27.1 % and over four household size accounted for 57.0 % as shown in Table 5. Lugaga/Wamuluma ward recorded the highest number of individuals who were more than three in the homestead, while Central Maragoli recorded the lowest (Table 5). Salary of the head of the homestead and household sizes in homesteads which varied in different wards tended to have had an effect on malaria prevalence in the study area (Table 5).

**Table 5:** Salary Distribution of Head of Household and Household Size in Different Geographical Locations (Wards) in the Study Area

	Geographical Locations (Ward)			
	Central Maragoli	Edzava	Lugaga/Wamuluma	Total
**Salary**				
High	30	53	76	159
Moderate	70	151	174	395
Low	44	74	96	214
**Total**	**144**	**278**	**346**	**768**
**Household size**				
Two	9	15	12	36
Three	19	32	35	86
Four	33	73	102	208
More than four	83	158	197	438
**Total**	**144**	**278**	**346**	**768**

**Salary:** (Low below Ksh. 10,000/month, Moderate Ksh. 10,000-20,000/month, High over Ksh.20, 000/month)

**Figure 2:**
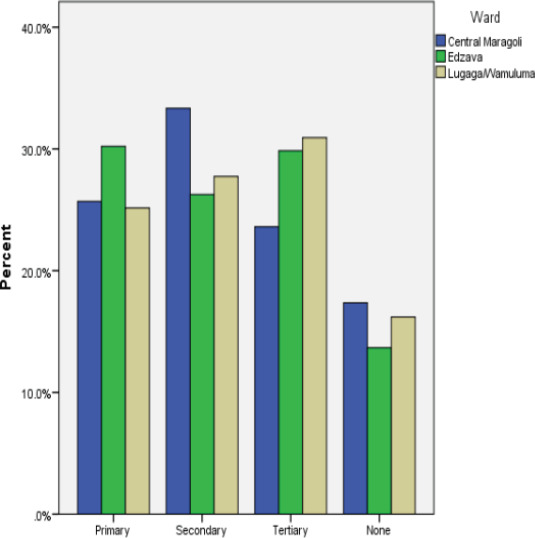
The Head of Household's Level of Education in Wards Found in the Study Area

Distribution of the head of the household's level of education in the study area was as follows; individuals who recorded primary education were 208 and no formal education 119 (below primary education) which accounted for 42.57% of the total study population, secondary education accounted for 217 (28.25%) and tertiary education accounted for 224 (29.16%) as shown in Figure 2. Lugaga/Wamuluma ward recorded the highest number of individuals in all levels of education (Individuals who recorded below secondary education accounted for 43.7%, secondary education 44.2% while individuals who recorded tertiary education accounted for 47.8%) as shown in Figure 2. Central Maragoli recorded the following results; individuals who recorded below secondary education accounted for 18.96%, secondary education 22.1% while individuals who recorded tertiary education accounted for 15.1%. In Edzava wards, individuals who recorded below secondary education accounted for 37.3%, secondary education 33.6% while individuals who recorded tertiary education accounted for 37%. Formal education tend to have an effect on malaria management as it enables individuals to understand the importance of malaria prevention and treatment.

**Figure 3:**
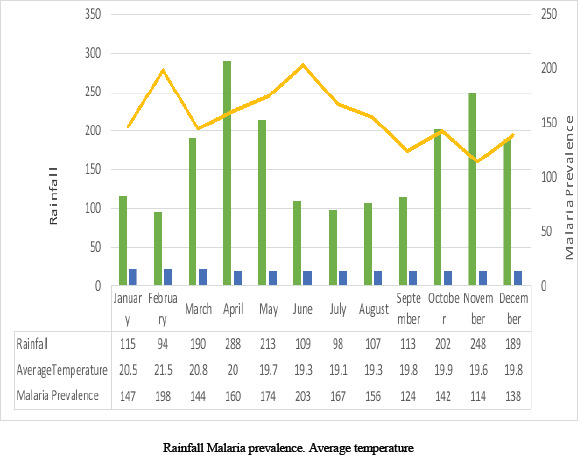
Effect of Time Trend on Malaria Prevalence in the Study Area

**Source:** Mbale Provincial Rural Training Health Center

Effect of rainfall on malaria prevalence was observed 1-2 months after the rainfall (Figure 3). High rainfall observed in April in the study area might have led to high malaria prevalence that was observed in June while low temperature and low rainfall in July was likely to have resulted to comparatively low malaria prevalence in September (Figure 3).

## Discussion

Characterization of spatial determinants and time-trends influenced malaria prevalence in the study area. From the linear regression analysis obtained, independent variables (wards, age and gender) showed the following effect on malaria prevalence (R2 = 0.7, [F (3,764) = 1.854], p < 0.136). Wards (space) had a statistically significant effect on malaria infection as opposed to gender and age in the study area. Distribution of *Plasmodium* species in the study area were as follows; *P. falciparum* malaria constituted 98.7% while *P. malariae, P. vivax* and *P. ovale* constituted 1.3%, which varied in gender, age groups and wards. Edzava ward recorded all the four Plasmodium species, while Central Maragoli and Lugaga/Wamuluma did not record *P. ovale* and *P. vivax* malaria. From the results obtained there were slightly more female malaria patients than male malaria patients^34^. *Plasmodium falciparum* malaria was the most common type of malaria among all age groups. Children aged 14-18 years old recorded *P. vivax* and recorded the highest *Plasmodium falciparum* malaria in the study area. From linear regression analysis, socio-economic factors had a statistically significant effect on malaria prevalence in the study area, (R2 = 0.061, [F (7,760) = 7.063], p < 0.000). The following socio-economic factors that were statistically significant to malaria prevalence in the study area; level of education, wealth, size of land, house type and ventilation in the house as opposed to salary and household size. Temperature and rainfall had influence on malaria prevalence in the study area. Heavy rainfall provided many breeding grounds for mosquitoes.

Socio-economic factors which tended to determine malaria transmission and to some extent influenced economic factors practiced, varied from one homestead to another in the study area. All the three wards found in study area were identified with various economic activities which included; crop farming, fish ponds, pits (tyre tracks) and old vehicle tyres found at car washing areas which provided many breeding grounds for *Anopheles* mosquitoes resulting to high malaria prevalence. Lugaga/Wamuluma ward recorded more malaria patients than the other two wards. *P. falciparum, P. vivax, P. ovale* and *P. malariae* vary in space and time trend (1). Malaria patients in Lugaga/Wamuluma ward were infected with mainly *P. falciparum* and 3 patients infected with *P. malaria. P. ovale* and *P. vivax* malaria were not identified in Lugaga/Wamuluma and Central Maragoli ward. In Central Maragoli ward malaria patients were mainly infected with *P. falciparum* and one patient recorded *P. malariae* malaria. All the four *Plasmodium* species (*P. falciparum P. malariae, P. ovale* and *P. vivax*) were recorded in Edzava ward. High malaria prevalence in Lugaga/Wamuluma ward was likely to have been due to poor drainage system and poor disposal of empty containers which encouraged collection of stagnant water providing breeding grounds for mosquitoes, high household size which might have compromised malaria management noting that poverty level and dependence ratio is very high in Vihiga County where the study area falls. In Lugaga/Wamuluma ward there was Ehedwe valley with low and gentle slopes, streams, bushes, swamps, pits formed by; car washing tyres, brick - making and gold mining which might have encouraged breeding of mosquitoes resulting to high malaria prevalence. In Edzava ward, high malaria prevalence was likely to have been caused by rain water that collected in the burrow pits formed by brick-making and gold mining which provided breeding grounds for mosquitoes. Central Maragoli ward was comparatively well drained and had few rocks which explained why malaria prevalence was fairly low compared to Lugaga/Wamuluma and Edzava wards. Results obtained from the study indicated that, in Lugaga/Wamuluma ward, 46.48% of the study population was unable to identify more than one mosquito breeding grounds while 35.77% of study population in Edzava and in Central Maragoli 17.74% of the study population could not correctly name more than one mosquito breeding grounds. This could have contributed to high malaria prevalence in the study area

Geographical locations (wards) which recorded many mosquito breeding grounds in the study area also recorded high malaria prevalence similar to findings in various parts of the Kenya and sub-Saharan Africa^5^,^8^. *Plasmodium falciparum* malaria was the most common malaria in the study area because it occurred during low and high rainfall seasons similar to findings in other regions in Kenya, Columbia and the world^4^, ^8^ ^35^. *Plasmodium falciparum* malaria prevalence in the study area accounted for 98.7% contrary to 99% reported in other parts of Kenya and in other parts of the world^1^,^5^,^8^. There were more malaria prevalence in female than male in the study area which was likely to have been due to socio-cultural norms that exposed female and children to mosquito bites, which was consistent with study findings that were carried out in Kenya on gender and malaria^34^. Children aged 14-18 years who were mainly primary and secondary school going children recorded high *Plasmodium falciparum* malaria compared to other age groups because they were more exposed to mosquito bites which was consistent with findings from other parts of the world^35^,^36^. *Plasmodium* species occurrence and *P. falciparum* infection varied by age which was consistent with findings in Burkina Faso^37^,^38^.

Socio-economic factors which were considered to be risk factors in malaria transmission in the study varied in different geographical location (wards) which to some extent influenced malaria prevalence in the study area supporting study findings from other parts of the world 1. Study population who recorded large number of assets accounted for only 25.9 % with Lugaga/Wamuluma ward leading in all categories of salary levels as Central Maragoli recorded the lowest in all salary categories. Poverty level in study area was high where 74.1 % of study population earned salary below Ksh. 20,000/month, which was likely to have made it difficult for study population to engage effectively in malaria management which was similar to study findings in other regions in Kenya and in sub-Saharan Africa countries^1^, ^2^ ^3^. High level of poverty in the study area was likely to have resulted to shortage of income to construct decent houses with ceiling boards to prevent entry of mosquitoes into the house and to seek medication in good time. 57 % of homesteads recorded more than four family members irrespective of study population's residence (ward) and the head of the homestead's level of education, which in addition to low income, might have resulted to such individuals to have faced challenges in purchasing adequate insecticides bed nets to supplement what the government had provided and unable to spray their houses with insecticides which exposed them to mosquito bites increasing malaria prevalence which supported study findings from other parts of the world^1^, ^8^. It was also observed that 46.2% of the study population, did not link malaria to more than one source of stagnant water which was a mosquito breeding ground but instead linked it to eating first harvest of maize and beans and as a curse from God. Study population in some rural parts of the study area rejected green and blue mosquito bed nets which they claimed suffocated them and led to miscarriage in pregnant mothers and instead used them to cover caught fish and to fence off vegetables gardens. Poor perception and knowledge on malaria management was common among individuals with low income and low education level similar to findings in other parts of Kenya^31^, and other parts of the world^1^. This was likely to have increased malaria prevalence in the study area.

It was observed at the health facility used that, adult malaria patients were more than malaria patients below 5 years old because most parents or guardians were reluctant to take their children to health facilities during COVID-19 pandemic period. Generally there should have been more malaria patients among children below 5 years old than adults but the study was purposive that is why equal number of malaria patients was taken from each age group. *Plasmodium* species occurrence and *P. falciparum* infection varied by age which was consistent with previous findings in Malawi and Burkina Faso^14^,^37^,^38^. Children below five years old and old people have low immunity levels and that is why they are easily infected with diseases, including malaria^1^, ^36^. Immunity to various diseases including malaria is high between people who are 10-50 years old^37^, ^38^. Children aged 5-14 years who were mainly nursery and primary school going children recorded all *Plasmodium* species apart from *P. vivax* while adults and children below 5 years old did not record *P. ovale*.^37^. Children between 14-18 years old who were mainly secondary and tertiary going children had more exposure to mosquito bites which enabled them to develop more immunity to malaria^36^,^37^. Children below 15 years old who include school-age children tend to acquire *P. falciparum* malaria easily according to findings from Burkina Faso^37^, which was contrary in the study area because *P. falciparum* malaria was more among children between 14-18 years old as compared to other age groups.

Temperature and rainfall had influence on malaria prevalence in the study area. There were many mosquito breeding grounds during heavy rainfall which was consistent with the study findings carried out in Vihiga County, Western Kenya, Malawi and other parts of the world^9^, ^12^, ^13^, ^14^. Heavy rainfall tends to wash away mosquito breeding sites hence reducing malaria prevalence following heavy rainfall^39^. In the study area, moderate rainfall tended to increase mosquito abundance which resulted to high malaria prevalence which was consistent with study findings from Nepal and other parts of the world^1^, ^16^. As monthly temperature increased the incidence rate of malaria increased significantly in the study area because increase in temperature tended to accelerate the development of malaria parasites in the *Anopheles* mosquitoes which was similar to findings in other regions of Western Kenya and in Kerman, South East of Iran^11^, ^39^. In the study area, malaria prevalence varied in different months due to changes in amount of rainfall received and temperatures which was consistent with findings from Kenya and other regions in the world^9^, ^12^, ^25^.

In the study area, effect of rainfall on malaria prevalence was observed 1-2 months after the rainfall season, because time taken from infective bite by *Anopheles* mosquitoes to appearance of the first symptoms of malaria is 7 to 30 days depending on the *Plasmodium* species, similar to study findings from Iran and other regions in the world^1^, ^39^. There was high malaria prevalence following long rains season and relatively high temperature in the study area which enabled mosquitoes to breed in many sites of stagnant water, developed very fast and accelerated the development of malaria parasites in *Anopheles* mosquitoes which was similar to findings carried out in Western Kenya, Nepal and Thailand^12^,^16^,^17^. There were more malaria prevalence in the month of June which was likely to have been due to the heavy rainfall that was observed in April which enabled the mosquitoes to live longer and increased malaria prevalence similar to study findings carried out in Ethiopia, Sudan and Iran^18^, ^20^, ^39^. In the study area, malaria prevalence was low in September which was likely to have been due to less rainfall and low temperature that were observed in July similar to study findings carried out in rural Western Kenya^24^. Malaria management bodies should closely monitor malaria prevalence patterns in relation to geographical regions at different time trend when implementing malaria control strategies in order to reduce malaria prevalence which is consistent with findings from Tanzania and Rwanda paying specially attention to individuals with low socio-economic status^21^,^22^.

## Conclusion and Recommendations

Spatio-temporal characterization influenced malaria prevalence in the study area. The study area recorded all the four *Plasmodium* species. Many mosquito breeding grounds created by various economic activities practised, topography, climate and environmental factors in the study area resulted to high mosquito abundance that led to high malaria prevalence. High poverty level, low salary and big household size in the study area also contributed to high malaria prevalence. Results from the research showed community health workers assisted in reducing cases of malaria mortality by testing and treating malaria patients especially young children at their homes. The Kenyan government should carry out regular monitoring and evaluation on influence of space and time trend on malaria prevalence patterns in order to accurately implement cost-effective malaria control strategies. The Kenyan government should also engage her people in poverty eradication programs in order to reduce and finally eliminate malaria.
